# Magnetic Properties of Ferritin at Different Levels of Degradation: Implications for MRI‐Based Iron Quantification in the Brain

**DOI:** 10.1002/mrm.70241

**Published:** 2025-12-31

**Authors:** Stefan Ropele, Sowmya Sunkara, Snježana Radulović, Saška Lipovšek, Michael Stöger‐Pollach, Christoph Birkl, Walter Gössler, Christian Enzinger, Gerd Leitinger

**Affiliations:** ^1^ Department of Neurology Medical University of Graz Graz Austria; ^2^ Division of Cell Biology, Histology and Embryology, Gottfried Schatz Research Center Medical University of Graz Graz Austria; ^3^ Faculty of Medicine University of Maribor Maribor Slovenia; ^4^ Faculty of Chemistry and Chemical Engineering University of Maribor Maribor Slovenia; ^5^ University Service Centre for TEM Technical University Vienna Vienna Austria; ^6^ Institute for Solid State Physics Technical University Vienna Vienna Austria; ^7^ Department of Radiology Medical University of Innsbruck Innsbruck Austria; ^8^ Institute of Analytical Chemistry Karl‐Franzens University Graz Austria

**Keywords:** degradation, ferritin, iron, postmortem, R_2_*, validation

## Abstract

**Purpose:**

Ferritin's iron core exhibits complex magnetic properties, as suggested by magnetometry and Mössbauer spectroscopy, which remain incompletely understood. In particular, the antiferromagnetic inner core could influence the accuracy of iron quantification using MRI and raise concerns about postmortem validation studies involving degraded ferritin cores.

**Methods:**

Fresh postmortem brain samples from six deceased human subjects were analyzed using energy‐filtered transmission electron microscopy (EFTEM) and electron energy loss spectrometry in scanning mode of the TEM (STEM‐EELS) to visualize and quantify the iron cores of ferritin proteins and estimate their iron content. EFTEM findings were compared with results from mass spectrometry and R_2_* mapping at 3T. Analyses focused on three gray matter regions including the frontal cortex, putamen, and globus pallidus.

**Results:**

Autolysis led to a rapid degradation of ferritin molecules, with fewer than one‐third remaining detectable via EFTEM 24 h postmortem. The degradation followed a single‐exponential decay pattern, suggesting that almost the entire non‐heme iron is stored in ferritin. However, R_2_* relaxation rates did not follow this degradation pattern but instead correlated strongly with total iron content as measured by mass spectrometry.

**Conclusion:**

R_2_* mapping‐derived magnetic susceptibility of ferritin appears to be independent of the structural and magnetic organization of its iron core and shows a linear relationship with total iron content. These findings support the interpretation of ferritin as a simple paramagnet at room temperature, without significant antiferromagnetic contributions. Consequently, susceptibility based postmortem studies focusing on iron accumulation are not affected by autolysis.

## 1 Introduction

Ferritin is a ubiquitously expressed protein and serves as the primary cellular iron storage molecule in the human body [1]. It consists of a globular protein shell that encapsulates up to 4500 Fe^3+^ ions in a soluble and non‐toxic form. This secure storage is crucial, as free iron ions can catalyze the production of free radicals and reactive oxygen species, potentially contributing to inflammatory and neurodegenerative diseases. The role of ferritin in these conditions remains an area of ongoing research [2]. Consequently, non‐invasive iron quantification using MRI has gained significant interest in recent years.

Ferritin is the most relevant non‐heme iron compound in the human body. Due to its paramagnetic properties, it can be effectively quantified using MRI. In the brain, where iron is the most abundant trace element [3], assessment of ferritin concentration typically focuses on deep gray matter, where diamagnetic contributions from myelin are minimal and where iron concentrations are highest. Several quantitative MRI parameters, including T_1_ and T_2_ relaxation times, have been shown to depend on iron concentration [4, 5, 6, 7]. However, R_2_* mapping and quantitative susceptibility mapping (QSM) are currently the most sensitive and reliable MRI methods for in vivo iron quantification [8, 9].

To achieve precise MRI‐based ferritin quantification in tissue, an appropriate and realistic magnetic model is required. Without such a model, disease induced variations in iron load or mineral core structure may introduce biases in iron quantification. For instance, studies have shown that co‐aggregation of amyloid‐beta and ferritin can lead to the reduction of ferritin's ferric iron core into more reactive low‐oxidation states, potentially contributing to neurotoxicity in Alzheimer's disease [10]. Two primary models have been proposed for the ferritin mineral core: a basic model, which assumes a simple paramagnetic behavior where each Fe^3+^ ion contributes additively to the bulk magnetic susceptibility [11], and a more advanced model, informed by magnetometry [5, 12] and Mössbauer spectroscopy [13]. The latter describes an antiferromagnetic inner core below the Néel temperature with incomplete cancellation of sublattices, surrounded by a highly disordered outer core exhibiting paramagnetic properties. These two models predict different susceptibility values at physiological temperatures for the same iron concentration.

This study aimed to challenge the current magnetic models of ferritin by investigating its magnetic properties at various levels of degradation. Furthermore, we sought to determine whether postmortem validation studies for iron mapping might be affected by ferritin degradation and the associated changes in its magnetic properties. To address this, we extended a recent transmission electron microscopy (TEM) study where we examined the cellular distribution of ferritin and the impact of autolysis on its detectability [14]. In the present work, we further employed advanced TEM techniques to estimate the mean size of the ferritin core. Combined with visual quantification of ferritin particles, this approach enabled a more quantitative assessment of degradation, which we then related to R_2_* measurements and total iron content.

## 2 Methods

### 2.1 Brain Samples

Fresh brain samples with postmortem intervals ranging from 6.5 to 24 h were obtained from six deceased human subjects undergoing routine brain autopsy. To minimize age‐related confounding factors, samples were collected exclusively from individuals over 60 years old, as natural iron accumulation saturation is typically reached in all brain structures by this age [15]. One hemisphere was preserved for R_2_* mapping, while regions from the contralateral hemisphere were dissected for mass spectrometry (MS) and transmission electron microscopy (TEM). The selected brain areas for this study‐ frontal gray matter (FGM), putamen, and globus pallidus—were chosen for their varying iron concentrations. After dissection, samples were fixed or placed in plastic tubes, weighed, and frozen for mass spectrometry analysis. Brain hemispheres designated for R_2_* mapping remained unfixed. The quantification of iron concentration with MS and the TEM‐based counting of ferritin have been described elsewhere [14]. The Ethics Committee of the Medical University of Graz approved this study (approval number 28‐549 ex 15/16).

### 2.2 MRI

MRI of the six unfixed brain hemispheres was performed on a 3 Tesla scanner (MAGNETOM PRISMA fit, Siemens Healthineers, Erlangen, Germany) using the standard 20 elements head coil for signal reception. The brain hemispheres were stored in a refrigerator but were allowed to reach room temperature before MRI. To obtain R_2_* relaxation data, a 2D RF spoiled multi‐echo gradient‐echo sequence with 6 equally spaced echoes was performed (repetition time = 1150 ms, first echo time = 4.92 ms, echo spacing = 5.42 ms, flip angle = 15°; field of view = 256 × 256 mm^2^; in‐plane resolution = 0.75 × 0.75 mm^2^; 2.4‐mm‐thick sections covering the entire hemisphere). To reduce phase dispersion effects that might affect R_2_* mapping and to increase B_0_ homogeneity, second‐order shimming was applied. To minimize susceptibility artifacts from remaining air bubbles, the brain samples were immersed in Galden SV80 (Solvay Plastics). R_2_*maps were calculated from the six echoes assuming a mono‐exponential decay. For a better identification of the specified regions of interest, a fast 2D spin echo sequence with the same spatial resolution and slice orientation was acquired.

### 2.3 TEM‐Based Quantification of Ferritin

Visual counting of iron‐loaded ferritins was done by four independent raters (SS, SR, SL, GL). Ferritins were only counted if they were clearly visible as a bright spot on both the iron L–jump‐ratio and the iron L–elemental maps and if at least three of the four raters agreed on their number [14]. If more than 20 ferritins were reported in any map, the mean value of the counts from every rater was taken. In total, 1080 iron L–elemental maps and 1080 iron L–jump‐ratios were generated and used for this study.

**FIGURE 1:**
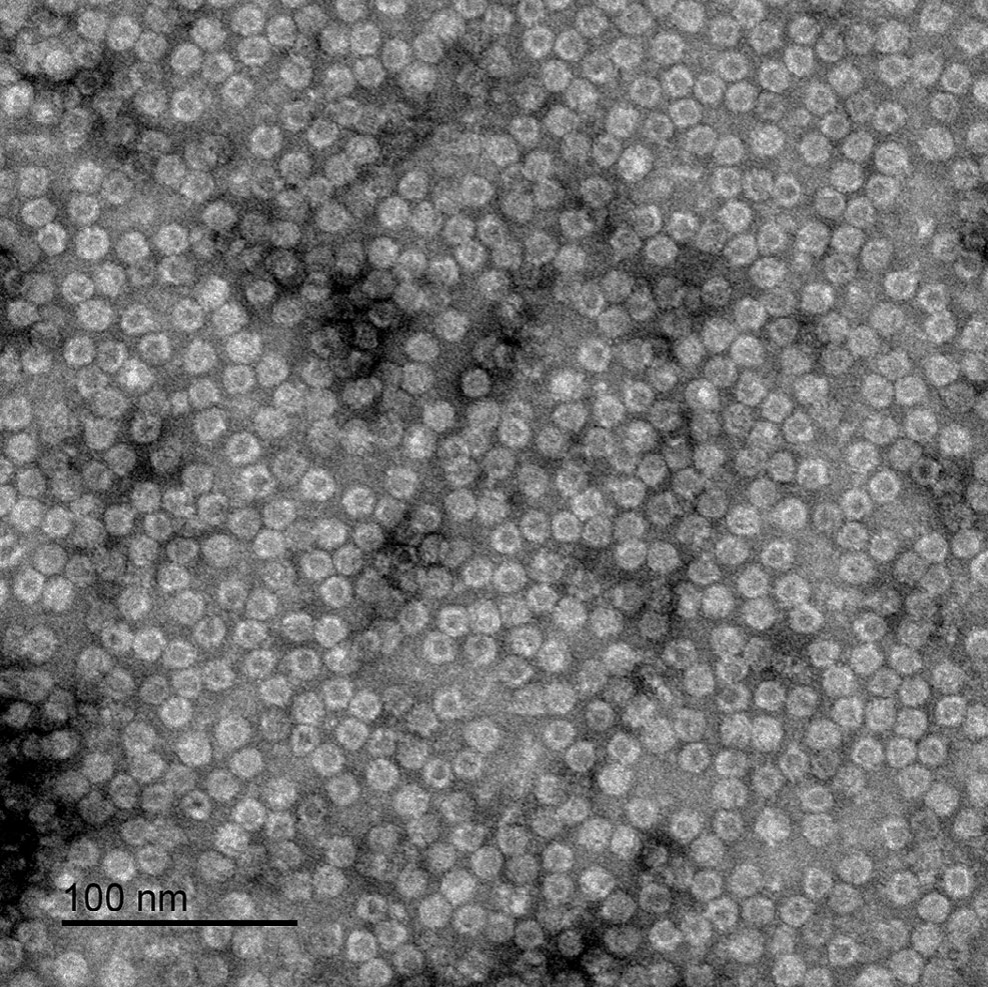
Negative contrast of isolated human ferritin from frontal cortex (PMI 20 h).

In order to determine the mean iron load of brain ferritin, ferritins from a sample with 20 h PMI were first isolated, placed on a carbon‐coated copper grid, and analyzed with EFTEM. The samples were homogenized with buffered protease inhibitor in a Turrax homogenizer IKA T‐10, centrifuged at 10 000 g for 30–60 min at 4°C and sonicated for 2 min at 4°C with a Branson Sonifier 250. The supernatant of another centrifugation step (10 000 g for 30–60 min at 4°C) was further purified: first by heating to 70°C–75°C for 10 min under constant stirring, then by precipitating the proteins in 75% saturated ammonium sulfate overnight at 4°C. The precipitated proteins centrifuged at 10 000 g for 60 min at 4°C, followed by size exclusion chromatography and density gradient centrifugation. Isolated ferritin was stained with 1% uranyl acetate to negatively contrast the sample before visualization by TEM (Figure 1).

To estimate the mean number of Fe atoms per ferritin, electron energy loss spectrometry in scanning mode of the TEM (STEM‐EELS) was employed. Thirteen ferritin particles were analyzed with a FEI Tecnai TF20 with high‐brightness electron source and a GATAN Tridiem image filter. The number of Fe atoms/nm^2^ was calculated by [16]

**FIGURE 2:**
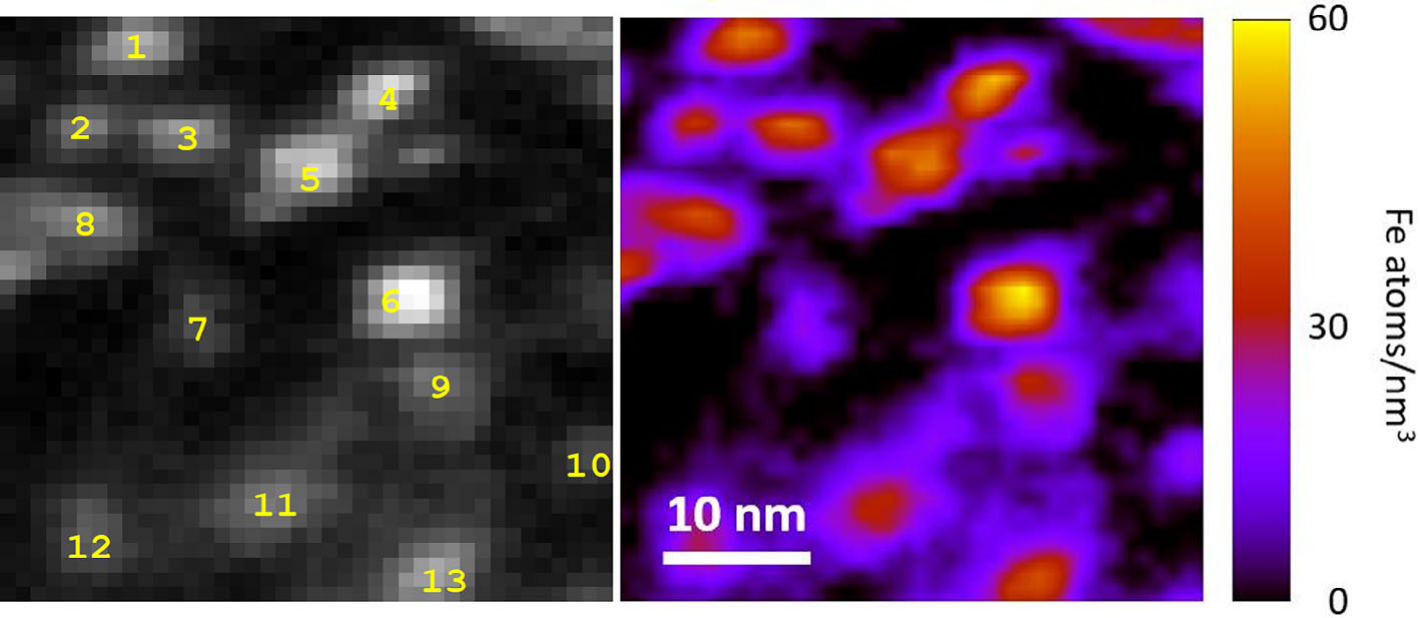
The diameter of 13 ferritin cores was measured from the STEM image (left) to calculate the atomic density of Fe (right). Randomly chosen extraction from the sample with isolated ferritin shown in Figure 1.

(1) NFeatomsnm2=IFeI0·1σFe·e−dλ

where *I*_Fe_ is the intensity in the Fe‐L edge, *I*_0_ is the total incoming intensity of the electron beam, *σ*_Fe_ is the ionization cross section of Fe for 200 keV electrons, *d* is the diameter of the ferritine core and *λ* is the mean free path length for inelastic scattering. Calculating the total amount of Fe atoms in a single ferritin molecule requires therefore knowledge about its shape. In the present analyses we assumed a spherical shape with diameter *d* being measured from the STEM image (Figure 2). The diameter was measured with the “particle analysis” plug‐in for DigitalMicrograph (Gatan Inc.), which is a histogram‐based tool for measuring sizes and radii from small particles with sufficiently high contrast.

The percentage of iron stored in ferritin was estimated by first calculating the total iron mass associated with ferritin. This was done by multiplying the average number of iron atoms per ferritin molecule by the atomic mass of iron (9.27 × 10^−20^ mg per atom), and then by the number of ferritin molecules observed per unit tissue volume. This ferritin‐bound iron mass was then expressed as a percentage of the total iron concentration in the tissue, as measured by MS. A brain tissue density of 1.076 kg/L was assumed to convert between mass and volume.

## 3 Results

**FIGURE 3:**
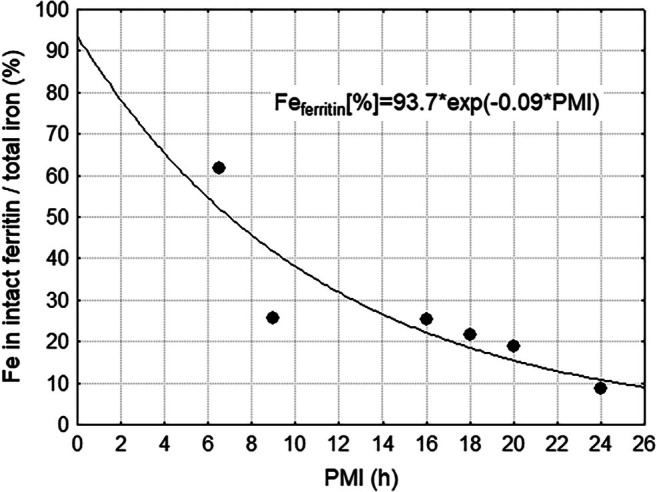
Percent of total iron stored in intact ferritin as a function of postmortem interval (PMI). Each filled circle represents the average value across the three gray matter regions from an individual subject. To avoid clutter from more than 6000 raw observations and amplified discretization at low counts, only the mean value is shown.

Visual analysis of TEM images indicated that ferritin cores in the postmortem brain undergo autolysis in all cellular structures, leading to the disappearance of approximately two‐thirds of all cores within the first 24 h postmortem. For the post mortem sample with 20 h PMI, quantitative STEM‐EELS analysis revealed a mean core size of 4.64 nm (min = 2.5 nm, max = 6.1 nm) and a mean load of 1740 (min = 370, max = 3604) Fe atoms per ferritin molecule (Table S1). To obtain normalized ferritin percentage for each gray matter region, the mean ferritin count was multiplied by the mean iron load and then related to the absolute iron content as assessed with MS for this region. The result is shown in Figure 3, which suggests an exponential degradation of the ferritin cores with a half‐life of approximately 8 h. An exponential decay curve appeared to be more appropriate (*R* ^2^ = 0.77) than a linear fit (*R* ^2^ = 0.71). It should be emphasized that the calculation is derived from intact ferritin cores detected by EFTEM. The appropriate interpretation is therefore an exponential decrease in the abundance of intact ferritin molecules, rather than a change in their mean iron load. The exponential fit also suggests that in living brain with a corresponding PMI of zero, more than 90% of all non‐heme iron is stored in ferritin.

**FIGURE 4:**
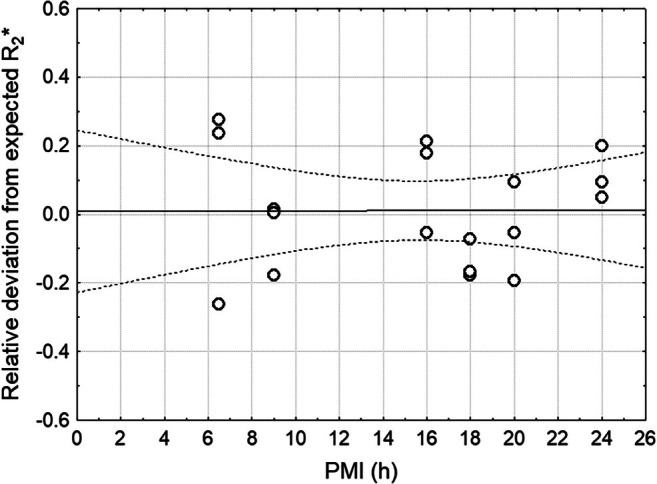
Effect of postmortem interval on R_2_* relaxivity. Shown is the relative deviation from the expected R_2_* value for each frontal and deep gray matter sample. The linear regression line indicates no measurable dependence on PMI. Dashed lines denote the 95% confidence interval.

**FIGURE 5:**
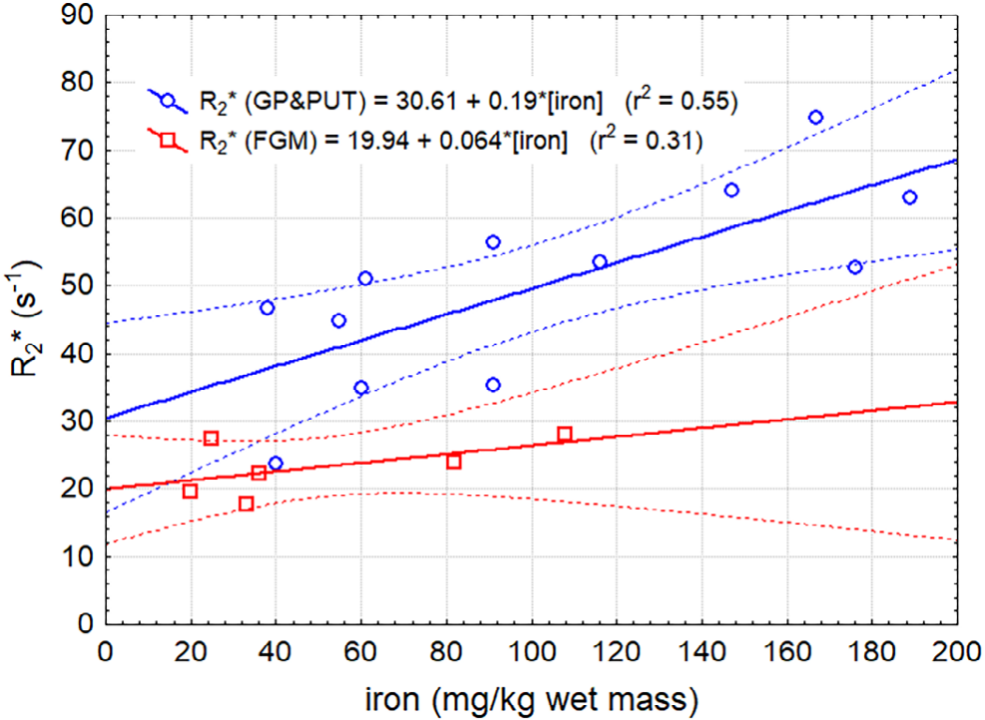
R_2_* shows a linear dependency from total iron concentration independent of the postmortem interval (FGM = frontal gray matter, GP = globus pallidus, PUT = putamen). Dashed lines represent the 95% confidence interval.

In contrast, R_2_* in FGM, putamen, and the globus pallidus did not show any significant change over time when considering individual variations in absolute iron content, suggesting that degradation of the ferritin did not affect its magnetic properties. Because longitudinal R_2_* measurements across PMIs were not feasible, we instead quantified the relative deviation of observed R_2_* values from those expected based on iron concentration, and tested whether these deviations depended on PMI using linear regression (Figure 4). Expected R_2_* values were derived from the regression relationship between R_2_* and absolute iron concentration across all samples (Figure 5). Even considering the substantial uncertainty, the lack of an exponential decay is clearly appreciable, especially when contrasted with Figure 3. When correlating R_2_* from all gray matter regions with the corresponding total iron concentration from MS, a strong and linear relationship was found, again indicating that R_2_* is directly related to iron concentration but not to the crystalline structure of the remaining iron core (Figure 5). In this analysis, the R_2_* relaxivity of iron was found to be lower in frontal cortex when compared to putamen and globus pallidus. A representative R_2_* map with the corresponding T_2_ weighted image is shown in Figure S1.

## 4 Discussion

While the structure of the ferritin core is usually considered to be based on that of the mineral ferrihydrite, the corresponding magnetic model at body temperature is not entirely clear yet. There is a lot of evidence from Mössbauer spectra and magnetometry that the inner core is antiferromagnetic with pairwise cancellation of most of the magnetic moments. However, most of these measurements have been done at very low temperatures and it is not totally clear if the Néel temperature, when the inner core becomes a paramagnet, is below body temperature. There are also considerable differences between human serum ferritin, brain ferritin and horse spleen ferritin with respect to heavy (H) chain and light (L) chain subunits and size of the core, but mostly horse spleen ferritin has been used for Mössbauer spectra and magnetometry so far. The magnetic configuration of the ferritin core remains highly relevant for in vivo mapping with MRI because most of the non‐heme iron in the human body is stored in ferritin. In models with antiferromagnetic ferritin cores and compensated moments, any process that affects the structure of the ferritin core would change its magnetic properties and in turn the relationship between iron concentration and magnetic susceptibility thus hampering precise quantification. The most obvious structural change of the ferritin core is due to autolysis. Validation studies for iron mapping can only be done postmortem and it can be expected that the ferritin structure differs from intact ferritin molecules in vivo. A contrary effect can be expected in vivo in another iron compound with unclear magnetic properties. Hemosiderin, an amorphous aggregate of denatured ferritin, iron, and other cellular debris, is typically found in conditions of iron overload or hemorrhage. Hemosiderin has no size limiting shell, is non‐spherical, and is usually much larger than ferritin which is why it can be seen under light microscope. Most assumptions regarding the crystalline structure and the corresponding magnetic properties of the aggregated iron have been forwarded from insights into ferritin but hemosiderin usually shows stronger inter‐particle interactions [17].

In this study, we provide further evidence that ferritin undergoes autolytic decomposition with an exponential course. Unlike the protein shell of ferritin, ferric iron (Fe^3+^) is usually considered poorly soluble when embedded in a crystalline structure. In living cells, ferritin is degraded in lysosomes which leads to the release of the stored iron. This process, known as ferritinophagy, involves the autophagic degradation of ferritin mediated by nuclear receptor coactivator 4 (NCOA4) [18]. While there is a lack of direct studies on postmortem ferritin degradation, it can be expected that similar acidic conditions arise during autolysis, where cellular components break down, potentially leading to ferritin destabilization and subsequent iron release. Our observations suggest that, over the course of the postmortem interval, the iron core either undergoes complete decomposition or fragments into smaller structures below the resolution of TEM. It should be noted that all calculations were based on the mean ferritin iron load from a single subsample. Because the isolation procedure (mass separation and then size‐exclusion chromatography) preferentially retains particles with intact cores and protein shells even at a PMI of 20 h, we expect the mean iron load of the intact ferritin fraction to show minimal variation with PMI. However, it cannot be ruled out that partially degraded ferritin may be underrepresented. If the exponential model of decomposition is valid, the resulting decay curve provides further insights into the distribution of iron compounds in the living brain. Specifically, the extrapolated value at zero postmortem delay in the exponential decay curve (Figure 3) represents the proportion of total iron stored in ferritin in vivo. Although direct quantification of ferritin in the brain is not feasible, previous studies suggest that at least 80%–90% of non‐heme brain iron is ferritin‐bound [4, 19]. Considering regional variations in non‐heme iron compound concentrations—due to differing metabolic and functional demands—as well as the uncertainty introduced by the limited sample size and interhemispheric differences, our estimate of 94% appears to be a reasonable approximation.

Despite the structural degradation of ferritin, the resulting magnetic effect detected via R_2_* mapping remains relatively stable throughout the entire postmortem interval and correlates well with total iron content. Interestingly, the R_2_* relaxivity of iron was found to be lower in the frontal cortex than in the putamen and globus pallidus. To our knowledge, this observation has not been reported previously. Although cortical tissue has a layered, complex microstructure and more restricted diffusion, the static dephasing regime does not account for the observed relaxivity difference. Partial‐volume effects with superficial white matter within cortical ROIs are a more plausible explanation. Nevertheless, the correlations suggest that the magnetic susceptibility is proportional to the total iron concentration and is largely independent of the ferritin core's loading factor or structural integrity. Although we could not directly probe the magnetic configuration of the ferritin core, our data favor a simple paramagnetic ferritin model. If the inner core were antiferromagnetic, its decomposition would be expected to increase magnetic susceptibility via release of ferric or ferrous ions, an effect not supported by our measurements. It should be noted that this study did not involve direct measurements of magnetic susceptibility. Instead, R_2_* mapping was employed as an indirect proxy. R_2_* reflects the sum of the transverse relaxation rate R_2_ and the rate R_2_′, which accounts for static dephasing caused by local magnetic field inhomogeneities. The field inhomogeneity in units of the Larmor frequency at the surface of a magnetic sphere is proportional to its total magnetization *μ* [20]

(2) $$\delta \omega =\mu \;\gamma /R^{3}$$

where *γ* is the proton gyromagnetic ratio, and *R* is the radius of the sphere. For sufficiently large particles composed of many paramagnetic ions, the total magnetization can be expressed by *M*_*0*_ as the magnetic moment per volume

(3) $$\mu =4\pi M_{0}R^{3}/3$$

Then, *δω* becomes proportional to *M*_0_ and independent of the size of the ferritin core. R_2_′ is linear proportional to the induced field inhomogeneity [21] and consequently a linear measure of the iron concentration. In contrast, R_2_ has a quadratic dependency on the field inhomogeneity [22], but with a much lower sensitivity for iron when compared to R_2_′. The relaxation rate R_2_* as the sum of R_2_ and R_2_′ is therefore expected to linearly scale with the total iron concentration, provided that there are no compensated magnetic moments in the inner core.

Our finding is further supported by evidence from a previous postmortem study [9], which included an even broader range of postmortem intervals and which yielded a temperature‐corrected susceptibility shift of 0.00097 ppm per mg iron per kg wet weight. This is very close to the predicted value from theory, when assuming solely paramagnetic ions in ferritin with each having an effective moment of 3.78 Bohr Magnetoms [11]. In case of large compensating moments in the inner core, a much lower mean effective moment would have been observed. In this context, more detailed insights might have been obtained through the use of QSM. However, a key limitation of the present study was the use of relatively thick slices in the gradient echo sequence, which may have also contributed to slightly elevated R_2_* values compared to those reported in previous studies [8]. Additionally, referencing relative susceptibility shifts was challenging due to the absence of cerebrospinal fluid or another suitable reference medium. Galden is proton‐free and therefore does not produce a MR signal, further complicating the QSM processing. However, R_2_* mapping is calibration‐free and more robust with fewer post processing steps involved.

## 5 Conclusion

Ferritin is the primary iron‐containing compound in the brain, accounting for approximately 95% of non‐heme iron. At room temperature, structural modifications within the ferritin core—whether due to autolysis or disease—do not appear to affect the magnetic properties of the associated iron. Consequently, the R_2_* relaxivity of Fe^3+^ remains stable. Overall, this study suggests a simple paramagnetic model for ferritin and also supports the validity of previous postmortem validation studies for iron mapping. These findings likely extend to hemosiderin and other iron compounds in the brain.

## Funding

This work was supported by the Austrian Science Fund (P29370).

## Supporting information


**Figure S1:** R_2_* map from unfixed brain tissue (right) and corresponding T_2_ weighted image (left). Manual outlining of the putamen (in blue) and frontal gray matter (in red) were done on the T_2_ weighted image. Note that only one hemisphere was available for MRI. Complementary analyses for these regions and also for the globus pallidus were done in the contralateral hemisphere.

**Table S1:** Diameter and number of Fe atoms in the ferritin core from the STEM‐EELS analysis. Core numbers shown here correspond to numbers in Figure 2.

## Data Availability

The data that support the findings of this study are available from the corresponding author upon reasonable request.

## References

[1] N. Zhang , X. Yu , J. Xie , and H. Xu , “New Insights Into the Role of Ferritin in Iron Homeostasis and Neurodegenerative Diseases,” Molecular Neurobiology 58, no. 6 (2021): 2812–2823, 10.1007/s12035-020-02277-7. 33507490

[2] T. A. Rouault , “Iron Metabolism in the CNS: Implications for Neurodegenerative Diseases,” Nature Reviews. Neuroscience 14, no. 8 (2013): 551–564, 10.1038/nrn3453. 23820773

[3] N. Krebs , C. Langkammer , W. Goessler , et al., “Assessment of Trace Elements in Human Brain Using Inductively Coupled Plasma Mass Spectrometry,” Journal of Trace Elements in Medicine and Biology 28, no. 1 (2014): 1–7, 10.1016/j.jtemb.2013.09.006. 24188895

[4] J. Vymazal , R. A. Brooks , C. Baumgarner , et al., “The Relation Between Brain Iron and NMR Relaxation Times: An in Vitro Study,” Magnetic Resonance in Medicine 35, no. 1 (1996): 56–61. 8771022 10.1002/mrm.1910350108

[5] R. A. Brooks , J. Vymazal , R. B. Goldfarb , J. W. M. Bulte , and P. Aisen , “Relaxometry and Magnetometry of Ferritin,” Magnetic Resonance in Medicine 40, no. 2 (1998): 227–235, 10.1002/mrm.1910400208. 9702704

[6] R. J. Ogg and R. G. Steen , “Age‐Related Changes in Brain T1 Are Correlated With Iron Concentration,” Magnetic Resonance in Medicine 40, no. 5 (1998): 749–753. 9797159 10.1002/mrm.1910400516

[7] K. M. Hasan , I. S. Walimuni , L. A. Kramer , and P. A. Narayana , “Human Brain Iron Mapping Using Atlas‐Based T2 Relaxometry,” Magnetic Resonance in Medicine 67, no. 3 (2012): 731–739, 10.1002/mrm.23054. 21702065 PMC3183376

[8] C. Langkammer , N. Krebs , W. Goessler , et al., “Quantitative MR Imaging of Brain Iron : A Postmortem Validation Study,” Radiology 257, no. 2 (2010): 455–462. 20843991 10.1148/radiol.10100495

[9] C. Langkammer , F. Schweser , N. Krebs , et al., “Quantitative Susceptibility Mapping (QSM) as a Means to Measure Brain Iron? A Post Mortem Validation Study,” Neuroimage 62, no. 3 (2012): 1593–1599, 10.1016/j.neuroimage.2012.05.049. 22634862 PMC3413885

[10] J. Everett , J. Brooks , F. Lermyte , et al., “Iron Stored in Ferritin Is Chemically Reduced in the Presence of Aggregating Aβ(1‐42),” Scientific Reports 10, no. 1 (2020): 1–16, 10.1038/s41598-020-67117-z. 32587293 PMC7316746

[11] J. F. Schenck , “The Role of Magnetic Susceptibility in Magnetic Resonance Imaging: MRI Magnetic Compatibility of the First and Second Kinds,” Medical Physics 23, no. 6 (1996): 815–850. 8798169 10.1118/1.597854

[12] L. Bossoni , J. A. Labra‐Muñoz , H. S. J. van der Zant , et al., “In‐Depth Magnetometry and EPR Analysis of the Spin Structure of Human‐Liver Ferritin: From DC to 9 GHz,” Physical Chemistry Chemical Physics 25, no. 40 (2023): 27694–27717, 10.1039/d3cp01358h. 37812236 PMC10583656

[13] T. G. St. Pierre , R. K. Pollard , D. DPE , R. J. Ward , and T. J. Peters , “Mössbauer Spectroscopic Studies of Deproteinised, Sub‐Fractionated and Reconstituted Ferritins: The Relationship Between Haemosiderin and Ferritin,” Biochim Biophys Acta (BBA) 952(C) (1988): 158–163, 10.1016/0167-4838(88)90111-2. 3337822

[14] S. Sunkara , S. Radulović , S. Lipovšek , et al., “Autolysis Affects the Iron Cargo of Ferritins in Neurons and Glial Cells at Different Rates in the Human Brain,” Cellular and Molecular Neurobiology 43, no. 6 (2023): 2909–2923, 10.1007/s10571-023-01332-w. 36920627 PMC10333380

[15] B. Hallgren and P. Sourander , “The Effect of Age on the Non‐Haemin Iron in the Human Brain,” Journal of Neurochemistry 3, no. 1 (1958): 41–51. 13611557 10.1111/j.1471-4159.1958.tb12607.x

[16] R. P. Ferrier , Electron Energy‐Loss Spectroscopy in the Electron Microscope, 3rd ed. (Springer New York, 2011), 10.1007/978-1-4419-9582-4.

[17] P. D. Allen , T. G. St Pierre , W. Chua‐Anusorn , V. Ström , and K. V. Rao , “Low‐Frequency Low‐Field Magnetic Susceptibility of Ferritin and Hemosiderin,” Biochimica et Biophysica Acta, Molecular Basis of Disease 1500, no. 2 (2000): 186–196, 10.1016/S0925-4439(99)00104-0. 10657588

[18] J. D. Mancias , X. Wang , S. P. Gygi , J. W. Harper , and A. C. Kimmelman , “Quantitative Proteomics Identifies NCOA4 as the Cargo Receptor Mediating Ferritinophagy,” Nature 508, no. 7498 (2014): 105–109, 10.1038/nature13148. PMC4180099 24695223

[19] C. M. Morris , J. M. Candy , A. B. Keith , et al., “Brain Iron Homeostasis,” Journal of Inorganic Biochemistry 47, no. 3–4 (1992): 257–265. 1431885 10.1016/0162-0134(92)84071-t

[20] P. Gillis and S. H. Koenig , “Transverse Relaxation of Solvent Protons Induced by Magnetized Spheres,” Magnetic Resonance in Medicine 345, no. 5 (1987): 323–345. 10.1002/mrm.1910050404 2824967

[21] D. A. Yablonskiy and E. M. Haacke , “Theory of NMR Signal Behavior in Inhomogeneous Tissues: The Static,” Magnetic Resonance in Medicine 32, no. 4 (1994): 749–763. 7869897 10.1002/mrm.1910320610

[22] Z. Gottesfeld and M. Neeman , “Ferritin Effect on the Transverse Relaxation of Water: NMR Microscopy at 9.4 T,” Magnetic Resonance in Medicine 35, no. 4 (1996): 514–520, 10.1002/mrm.1910350410. 8992201

