# Supplementary material for: Magnetic Properties of Ferritin at Different Levels of Degradation: Implications for MRI‐Based Iron Quantification in the Brain
Source: Magn Reson Med. 2025 Dec 31;95(5):2892–7. doi: 10.1002/mrm.70241 (PMC12962208; doi:10.1002/mrm.70241)
Supplement: Supplementary file 1 — Figure S1: R2* map from unfixed brain tissue (right) and corresponding T2 weighted image (left). Manual outlining of the putamen (in blue) and frontal gray matter (in red) were done on the T2 weighted image. Note that only one hemisphere was available for MRI. Complementary analyses for these regions and also for the globus pallidus were done in the contralateral hemisphere. [file MRM-95-2892-s001.docx]

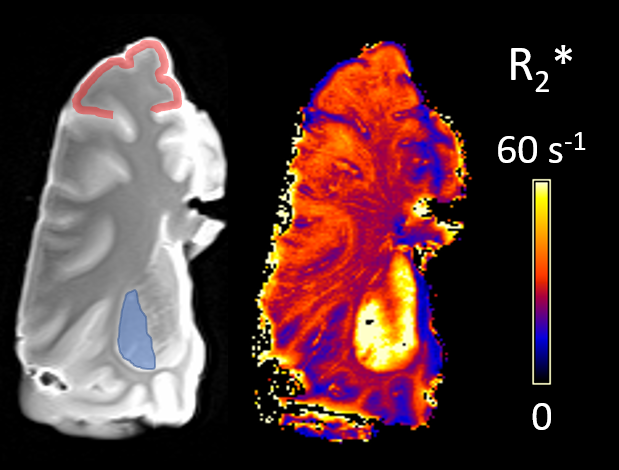


**Figure S1.** *R_2_* map from unfixed brain tissue (right) and corresponding T_2_ weighted image (left). Manual outlining of the putamen (in blue) and frontal gray matter (in red) were done on the T_2_ weighted image. Note that only one hemisphere was available for MRI. Complementary analyses for these regions and also for the globus pallidus were done in the contralateral hemisphere.*
