# Supplementary material for: Magnetic Properties of Ferritin at Different Levels of Degradation: Implications for MRI‐Based Iron Quantification in the Brain
Source: Magn Reson Med. 2025 Dec 31;95(5):2892–7. doi: 10.1002/mrm.70241 (PMC12962208; doi:10.1002/mrm.70241)
Supplement: Supplementary file 2 — Table S1: Diameter and number of Fe atoms in the ferritin core from the STEM‐EELS analysis. Core numbers shown here correspond to numbers in Figure 2. [file MRM-95-2892-s002.docx]

| **number** | **diameter** | **atoms/nm3** | **total # atoms** |
| --- | --- | --- | --- |
| 1 | 5.35 | 28.50 | 2285.47 |
| 2 | 4.20 | 26.19 | 1015.99 |
| 3 | 4.50 | 29.11 | 1388.98 |
| 4 | 4.90 | 31.94 | 1967.46 |
| 5 | 6.10 | 26.89 | 3195.23 |
| 6 | 6.10 | 30.33 | 3604.38 |
| 7 | 2.85 | 30.53 | 370.01 |
| 8 | 5.40 | 24.44 | 2015.39 |
| 9 | 4.80 | 23.96 | 1387.33 |
| 10 | 3.10 | 28.71 | 447.83 |
| 11 | 5.50 | 22.91 | 1995.70 |
| 12 | 3.95 | 26.84 | 865.96 |
| 13 | 5.50 | 25.27 | 2201.60 |

**Table S1.** *Diameter and number of Fe atoms in the ferritin core from the STEM-EELS analysis. Core numbers shown here correspond to numbers in Figure 2.*
